# Correlating p53 immunostaining patterns with somatic *TP53* mutation and functional properties of mutant p53 in triple‐negative breast cancer

**DOI:** 10.1111/his.15453

**Published:** 2025-03-31

**Authors:** Meejeong Kim, Miseon Lee, Ahwon Lee, Byung‐Ock Choi, Woo‐Chan Park, Sung Hun Kim, Jieun Lee, Jun Kang

**Affiliations:** ^1^ Department of Hospital Pathology, Seoul St. Mary's Hospital, College of Medicine The Catholic University of Korea Seoul Korea; ^2^ Cancer Research Institute The Catholic University of Korea Seoul Korea; ^3^ Department of Radiation Oncology, Seoul St. Mary's Hospital, College of Medicine The Catholic University of Korea Seoul Korea; ^4^ Division of Breast Surgery, Department of Surgery, Seoul St. Mary's Hospital, College of Medicine The Catholic University of Korea Seoul Korea; ^5^ Department of Radiology, Seoul Saint Mary's Hospital, College of Medicine The Catholic University of Korea Seoul Korea; ^6^ Division of Medical Oncology, Department of Internal Medicine, Seoul St. Mary's Hospital, College of Medicine The Catholic University of Korea Seoul Korea

**Keywords:** gain‐of‐function, mutant p53, p53 expression, p53 immunohistochemistry, TP53 mutation, triple‐negative breast cancer

## Abstract

**Aims:**

Immunohistochemical (IHC) staining of p53 is a potential marker for *TP53* mutations in various cancers. However, criteria for predicting *TP53* mutations in triple‐negative breast cancer (TNBC) using p53 IHC staining are not yet established. We aim to correlate p53 IHC expression patterns with *TP53* mutation status in TNBC.

**Methods and Results:**

A total of 113 TNBC cases were analysed for p53 IHC staining pattern and somatic *TP53* mutation using whole‐exome sequencing. Functional properties of *TP53* mutations were determined using the National Cancer Institute (NCI) *TP53* database. P53 IHC patterns were categorized as nuclear overexpression (*n* = 58), null pattern (*n* = 40), wildtype (*n* = 15), cytoplasmic (*n* = 5), and subclonal (*n* = 5). The cutoff for predictive p53 nuclear overexpression was determined to be 80%, which strongly correlated with *TP53* mutations. Notably, p53 overexpression had a positive predictive value (PPV) of 83% for missense or in‐frame mutations, while the null pattern showed a PPV of 85% for detecting nonsense, frameshift, or splicing mutations. P53 overexpression was significantly linked to missense mutations within the DNA‐binding domain (DBD) exhibiting gain‐of‐function (GOF) or dominant‐negative effect (DNE). Cases exhibiting cytoplasmic expression correlated with nonsense or frameshift mutations in the DBD, nuclear localization signal (NLS), or splice sites. Cases with subclonal p53 staining patterns were associated with *TP53* mutations.

**Conclusion:**

Our study proposes newly defined criteria for interpreting p53 immunostaining patterns in TNBC, potentially allowing for the prediction of *TP53* mutation types and their functional implications.

## Graphical abstract

This study introduces newly defined criteria for interpreting p53 IHC expression in triple‐negative breast cancer. These criteria could enable the prediction of *TP53* mutation types and their functional implications, providing valuable insights for future clinical research and potentially improving stratification of treatment options for patients.

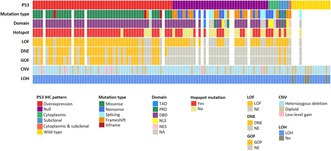

**Abbreviations**
AUC: area under the curve
CI: confidence interval
CNV: copy number variation
CTD: C‐terminal domain
DBD: DNA‐binding domain
DNE: dominant‐negative effect
ER: estrogen receptor
FFPE: formalin‐fixed, paraffin‐embedded
GOF: gain‐of‐function
IHC: immunohistochemistry
LOF: loss‐of‐function
LOH: loss of heterozygosity
NE: no evidence
NES: nuclear export signal
NLS: nuclear localization signal
NPV: negative predictive value
PPV: positive predictive value
PR: progesterone receptor
PRD: proline‐rich domain
ROC: receiver operating characteristic
SNP: single nucleotide polymorphism
TAD: transactivation domain
TNBC: triple‐negative breast cancer

## Introduction

Triple‐negative breast cancer (TNBC) represents a distinct subtype characterized by the absence of expression of oestrogen receptor (ER) and progesterone receptor (PR) and the lack of expression or amplification of human epidermal growth factor receptor 2 (HER2). It comprises a heterogeneous group of tumours with aggressive behaviour, limited treatment options, and a higher relapse risk.^1^ *TP53* mutations frequently occur in TNBC with a high prevalence (60%–80%), serving as potential therapeutic targets or predictors of chemotherapy response.^2^, ^3^, ^4^, ^5^

The *TP53* gene at 17p13.1, encodes the tumour suppressor p53, a key guardian of genomic integrity. P53 has four functional domains: the N‐terminal transactivation domain (TAD), the proline‐rich domain (PRD), the DNA‐binding domain (DBD), which is often the focus of mutations, and the unstructured C‐terminal domain (CTD) including a nuclear localization signal (NLS) and a nuclear export signal (NES).^6^ As a gene expression regulator, p53 manages stress responses like cell cycle arrest, apoptosis, and senescence.^7^ *TP53* mutations disrupt tumour‐suppressive function of p53, aiding cancer progression. Immunohistochemistry (IHC) of p53 is a sensitive and specific tool (>95%) for inferring *TP53* mutation status in cancers.^8^, ^9^

The relationship between *TP53* mutations and p53 IHC patterns is well‐studied in ovarian carcinoma, endometrial carcinoma, and vulvar squamous cell carcinoma but remains unclear in breast cancer.^10^, ^11^, ^12^, ^13^, ^14^ A specific threshold for p53 overexpression is not established, although recent studies suggest overexpression in at least 80% of tumour cells may predict *TP53* mutations.^15^, ^16^ The impact of *TP53* mutations on p53 gain‐of‐function (GOF) activities is not well understood, and the correlation between these mutations, p53 IHC patterns, and their functional effects remain unclear.^17^, ^18^, ^19^

This study aimed to define aberrant p53 IHC patterns indicative of mutation‐driven expressions associated with *TP53* mutations in TNBC and to elucidate the correlation between *TP53* mutation functions and aberrant p53 IHC expression patterns.

## Materials and Methods

### Case selection

This study included 113 consecutively collected cases of TNBC who underwent surgery at Seoul St. Mary's Hospital between 2018 and 2022. Approved by the Catholic University of Korea's Institutional Research Ethics Board (IRB No. KC22SISI0716), the study waived patient consent and followed the Declaration of Helsinki (2013 revision). Cases with neoadjuvant therapy or tumours smaller than 1.5 cm were excluded to reduce technical limitations associated with whole‐exome sequencing and enhance the reliability of the results. Ancillary test information for confirmation of TNBC is in Method S1. Histological classification followed the 5th edition of the WHO classification, and type, grade, and stage were reviewed using the 8th edition AJCC TNM classification.^20^

### P53 immunohistochemistry

Detailed procedures for p53 immunostaining are described in Method S2. One of four pathologists reviewed p53 IHC slides, blinded to *TP53* mutation status, assessing nuclear expression in tumour cells. Expression was quantified in 5% increments from 0% to 100%. Three additional pathologists reviewed the assessments, with discordant cases discussed until consensus was reached. A p53 expression rate‐dependent ROC curve was analysed using *TP53* mutation status to determine the optimal cutoff for p53 IHC overexpression based on the Youden index. A p53 expression rate‐dependent receiver operating characteristic (ROC) curve was analysed using *TP53* mutation status to determine the optimal cutoff for p53 IHC overexpression based on the Youden index.

### Whole‐exome sequencing

Whole exome sequencing was performed on formalin‐fixed, paraffin‐embedded tissue from 113 TNBC cases using the NovaSeq6000 (Illumina, San Diego, CA, USA) platform. Paired‐end sequences were aligned to the human genome (hg19) with Burrows–Wheeler, and somatic *TP53* mutations were identified using MuTect2 and annotated with SnpEff.^21^ Copy number and loss of heterozygosity (LOH) analyses were conducted using GISTIC 2.0 and GenomicRanges, focusing on significant loci and *TP53*‐specific LOH.^22^, ^23^ The detailed method for sequencing analysis, including data annotation, copy number, and LOH analyses, is provided in Method S3.

### Database for functional classification of TP53 mutation

Functional classification of *TP53* mutations was based on data from the NCI *TP53* database (R20 version; https://tp53.isb‐cgc.org), categorizing them as LOF, DNE, or GOF.^24^ Specifically, we considered LOF when the mutant protein resulted in theloss of functional properties of wildtype p53. DNE was considered when mutant proteins inhibited the wildtype protein in the transactivation or cell growth assays. GOF encompassed functional properties exhibited by the mutant protein but not by the wildtype counterpart. In the current study, a mutant p53 was classified as having GOF when it met at least one of the following categories: (1) tumorigenic property (in nude mice) in transfected cells; (2) interference with p73 activity; (3) transactivation of genes repressed by wildtype p53; (4) resistance to a cytotoxic drug; (5) increase growth rate; (6) cooperation with an oncogene for the transformation of rat embryonic fibroblast or mouse embryonic fibroblast cells; and (7) alteration of mutant p53 stability and activity by impairing regulators, including HSC70 and MDM2.^25^, ^26^

### Statistical analysis

The Youden Index determined the optimal cutoff for p53 overexpression, defined as sensitivity + specificity−1. The performance of this cutoff in predicting TP53 mutation was evaluated using AUC, sensitivity, specificity, positive predictive value (PPV), negative predictive value (NPV), and accuracy. The association between p53 IHC patterns and TP53 mutation types was analysed using chi‐square or Fisher's exact test, with significance set at *P* ≤ 0.05. All analyses were performed using R version 4.3.1 (R Foundation, Vienna, Austria).

## Results

### P53 immunohistochemistry patterns

P53 overexpression for *TP53* mutation prediction achieved an area under the curve (AUC) of 0.88 (95% confidence interval [CI]: 0.76–0.98). We established a new cutoff value for p53 overexpression at 80% to optimally predict *TP53* mutation, corresponding to the highest Youden index. This threshold achieved a sensitivity of 0.95 (95% CI: 0.88–0.99), a specificity of 0.78 (95% CI: 0.52–0.94), and an accuracy of 0.91 (95% CI: 0.84–0.97). We observed that an increase in p53 expression rate improved the accuracy of *TP53* mutation prediction, as evidenced by a rising Youden index, which peaked at 80% (Figure S1A,B). Cases with *TP53* mutations predominantly showed p53 nuclear expression rates above 75%, indicating a strong correlation between a high p53 expression rate and the presence of *TP53* mutations (Figure S1C).

**Figure 1:**
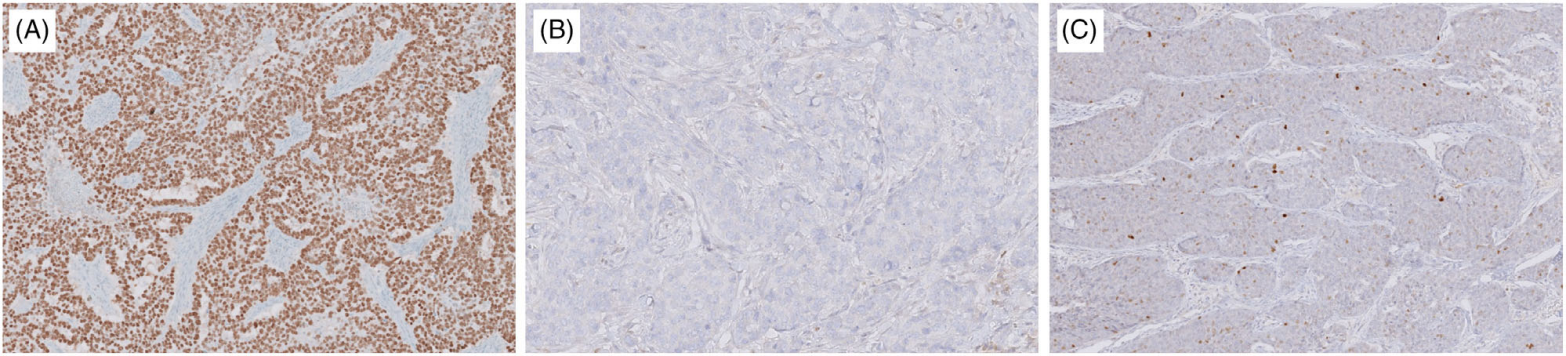
Representative patterns of p53 immunohistochemical staining in triple‐negative breast cancer. (**A**) Overexpression pattern of p53 in more than 80% of tumour cells with strong nuclear expression. (**B**) Complete loss of staining (null pattern). (**C**) Wildtype pattern with variable and patchy nuclear expression.

**Figure 2:**
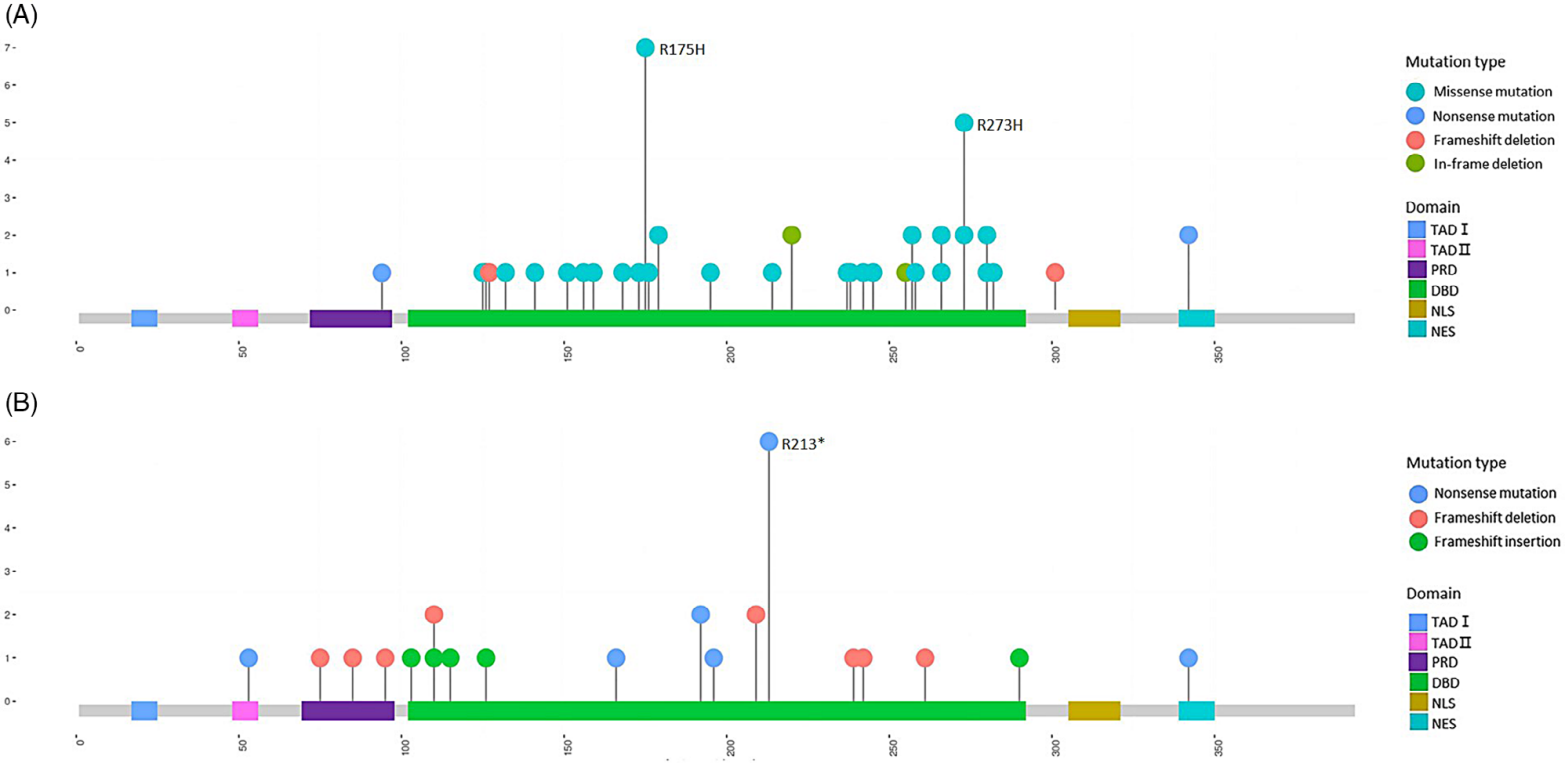
Lollipop plot depicting the frequency and position of *TP53* mutations based on p53 immunohistochemical staining patterns. Localization of *TP53* mutation in (**A**) p53 overexpression (*n* = 60) and (**B**) p53 null pattern (*n* = 40).

**Figure 3:**
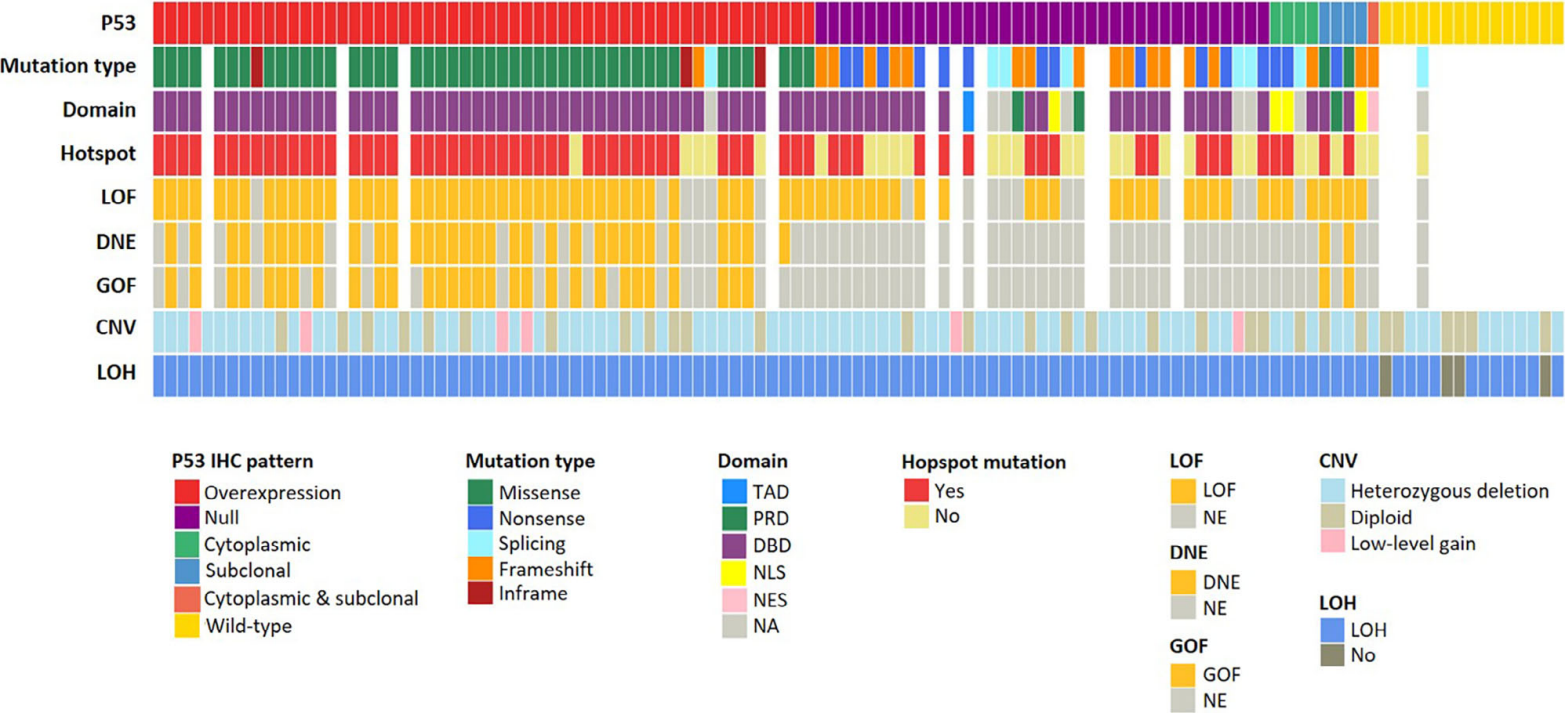
Distribution of p53 immunohistochemical staining patterns and *TP53* alteration in triple‐negative breast cancer. The oncoplot presents *TP53* status, including mutation parameters, functional properties, and copy number variations, according to p53 staining patterns. CNV, copy number variation; DBD, DNA‐binding domain; GOF, gain‐of‐function; LOH, loss of heterozygosity; NA, not available; NE, no evidence of functional property; NES, nuclear export signal; NLS, nuclear localization signal; PRD, proline‐rich domain; TAD, transactivation domain.

**Figure 4:**
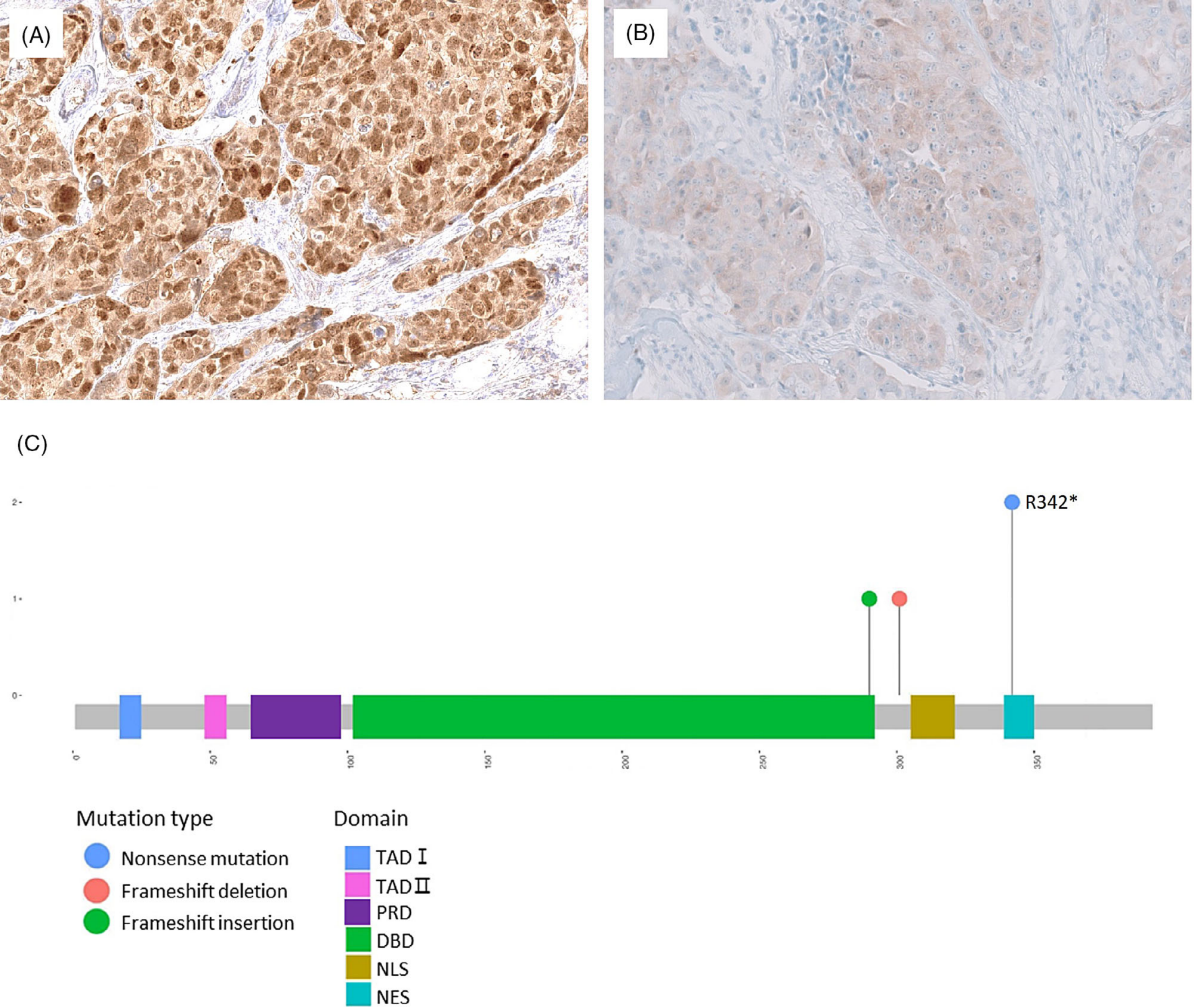
Cytoplasmic p53 staining patterns and a lollipop plot of *TP53* mutations. (**A**) Case with moderate to strong cytoplasmic and nuclear staining harbouring TP53 nonsense mutation, R342*. (**B**) Case with weak cytoplasmic expression and the absence of nuclear expression harbouring *TP53* frameshift deletion, S261Vfs*84. (**C**) *TP53* mutation frequency and position in the four cases with cytoplasmic expression, excluding one splicing mutation for which the domain location was not available.

IHC staining patterns of p53 were classified into five categories: (1) nuclear overexpression, characterized by strong and diffuse nuclear staining in at least 80% of tumour cells (Figure 1A); (2) complete absence of staining (null pattern; Figure 1B); (3) wildtype, identified by a mix of positive and negative cells at varying intensities, constituting less than 80% of tumour cells (Figure 1C); (4) cytoplasmic expression; and (5) subclonal expression. Cytoplasmic and subclonal expression patterns, identified in a subset of cases, are detailed in a subsequent section. In a total of 113 TNBC cases, p53 IHC expression patterns included overexpression in 58 cases, null in 40 cases, and wildtype in 15 cases. Clinicopathologic characteristics of patients are summarized in Table S1. Although established criteria for classifying p53 immunostaining patterns in breast cancer are lacking, these categories we presented were adapted from widely used standards in various cancers, particularly endometrial and ovarian carcinomas.^8^, ^11^, ^27^

### Correlations between p53 immunostaining patterns and TP53 alterations

Somatic *TP53* mutation was detected in 89 (79%) of 113 TNBC cases. Two cases exhibiting double mutations: R280T + G245D in one patient and p.S127Lfs*42 + splicing mutation in another patient. These two cases harbouring two types of *TP53* mutations simultaneously were counted as one for mutation type in the classification. Therefore, 91 *TP53* mutations were classified as missense (*n* = 47), in‐frame (*n* = 3), frameshift (*n* = 18), nonsense (*n* = 15), and splicing (*n* = 8) mutations. Types of *TP53* mutations according to p53 IHC patterns are presented in Table 1. We identified 11 discordant cases between IHC and sequencing results: 10 cases with aberrant p53 expression (overexpression, null, cytoplasmic, or subclonal) but no *TP53* mutation (four with overexpression and six with a null pattern), and one case with wildtype p53 expression despite a *TP53* mutation (Table 1).

**Table 1:** Correlation between p53 immunohistochemical staining pattern and *TP53* mutational status

P53 pattern	TP53 mutation status, *n* (%)						Total
	Missense	In‐frame	Frameshift	Nonsense	Splicing	Absent	
Overexpression	47 (78%)	3 (5%)	2 (3%)	3 (5%)	1 (2%)	4 (7%)	60^a^ (100%)
Null	0	0	16 (40%)	12 (31%)	6 (15%)	6 (15%)	40 (100%)
Cytoplasmic^b^	0	0	2 (40%)	2 (40%)	1 (20%)	0	5 (100%)
Subclonal^b^	2 (40%)	0	2 (40%)	1 (20%)	0	0	5 (100%)
Wildtype	0	0	0	0	1 (7%)	14 (93%)	15 (100%)

^a^ The total number is based on the count of 60 mutant variants in 58 cases, including two cases with double mutations: R280T + G245D, and S127Lfs*42 + splicing mutation.

^b^ All cases overlap with nuclear overexpression or null patterns, resulting in overlapping classification.

The PPV for p53 overexpression in detecting missense or in‐frame mutations was 83%. The PPV for p53 null pattern in predicting nonsense, frameshift, or splicing mutations was 85% (Table 2). The majority (91%, 51 of 56) of cases with p53 overexpression had *TP53* mutations in the DBD (Figure 2A). In contrast, 68% (23 of 34) cases with p53 null patterns showed DBD site mutations, which were fewer than those with p53 overexpression. Instead, cases with p53 null patterns had more frequent mutations at other sites: splice site (18%), TAD (3%), PRD (9%), and NES (6%) (Figure 2B).

**Table 2:** P53 immunostaining patterns and mutation type prediction

Statistics	P53 overexpression (missense/in‐frame)	P53 null pattern (nonsense /frameshift/splicing)
Sensitivity (95% CI)	100% (93–100)	85% (70–94)
Specificity (95% CI)	85% (74–92)	92% (83–97)
Positive predictive value (95% CI)	83% (74–90)	85% (72–93)
Negative predictive value (95% CI)	100% (94–100)	92% (84–96)
Accuracy (95% CI)	91% (85–96)	89% (82–94)

In 89 TNBC cases with *TP53* mutations, 63 (71%) exhibited heterozygous deletion and five (6%) showed low‐level gain. There were no cases of homozygous deletion or amplification. All 89 cases with *TP53* mutations had loss of heterozygosity (LOH), including 21 (23%) cases with copy neutral LOH. There was no significant relationship between p53 IHC pattern and *TP53* copy number variation (CNV) or LOH.

### Functional properties of mutant p53 and their correlations with p53 immunostaining patterns

Using the National Cancer Institute (NCI) *TP53* database, assessment of functional properties of mutant p53 was available for 72 of 91 *TP53* mutations. Functional properties were not reported for several mutations, including splicing mutations (*n* = 8), frameshift mutations (*n* = 6), in‐frame deletions (*n* = 3), nonsense mutation (*n* = 1), and missense mutation (*n* = 1). Consequently, these were classified into a no evidence (NE) group. Functional heterogeneity enabled a mutant p53 protein to concomitantly exhibit loss of function (LOF), dominant‐negative effect (DNE), and GOF as described in the Discussion section. All 72 *TP53* mutations were classified as LOF. Of these, 35 exhibited DNE, 31 of which also demonstrated GOF.

Figure 3 summarizes characteristics of *TP53* alteration and the associated mutant p53 function according to p53 IHC expression patterns in 113 TNBC cases. The majority of p53 overexpression exhibited GOF predominantly comprising missense mutations mainly located in the DBD, while none of the cases with null or wildtype pattern showed GOF (*P* < 0.001). Additionally, p53 overexpression (compared to null or wildtype pattern) was significantly associated with hotspot mutations (*P* < 0.001). GOF mutations were more likely to exhibit hotspots than NE‐GOF mutations (*P* < 0.001). GOF properties for 31 *TP53* mutations representing 17 unique mutations are summarized in Table S2. There was no relationship between the functionality of mutant p53 and CNV or LOH.

### Cytoplasmic or subclonal p53 immunostaining pattern with TP53 mutation

Apart from the p53 IHC pattern classification based on nuclear expression, a minority of cases exhibited cytoplasmic or subclonal staining patterns. These cases showed unique features of *TP53* mutations, which are separately detailed in our findings. Cytoplasmic p53 expression defined as at least 80% of tumour cells in this study with variable nuclear staining was observed in five (4%) of 113 of TNBCs, including three cases with p53 nuclear overexpression exhibiting a weak to moderate cytoplasmic staining pattern (Figure 4A) and two cases with a complete absence of p53 nuclear expression but weak cytoplasmic expression (Figure 4B). None of these cases showed cytoplasmic expression with a wildtype p53 nuclear pattern. All cases with a cytoplasmic pattern harboured *TP53* mutations, including nonsense mutations in the NES (*n* = 2), frameshift mutations in the DBD and NLS, and a splicing mutation (and Figure 4C).

**Figure 5:**
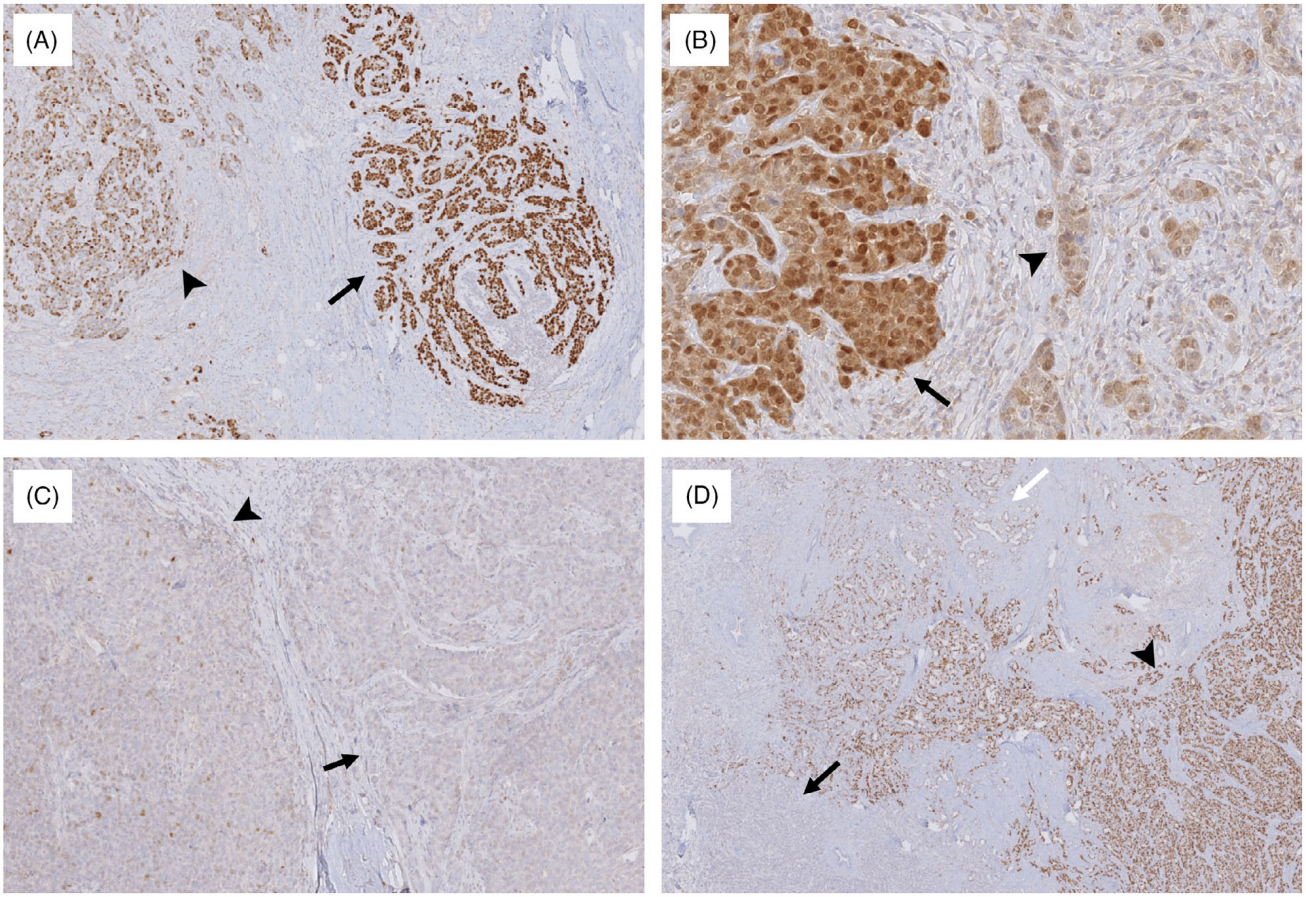
Representative cases with subclonal p53 staining patterns. (**A**) Case exhibiting a subclonal pattern with a combination of p53 overexpression (arrow) and wildtype pattern (arrowhead) with a *TP53* missense mutation, M237I in DNA‐binding domain. (**B**) Case displaying overexpression (arrow) and wildtype (arrowhead) patterns with cytoplasmic expression harbouring a *TP53* frameshift deletion, P301Qfs44, near the nuclear localization signal. (**C**) Case with a subclonal pattern showing a combination of wildtype (arrowhead) and null pattern (arrow) harbouring a *TP53* frameshift deletion, E343Gfs2, near the nuclear export signal domain. (**D**) Case with a mixture of wildtype (white arrow), null (black arrow), and overexpression (arrowhead) harbouring a *TP53* nonsense mutation, S94* near the proline‐rich domain.

We identified five cases with a subclonal p53 staining pattern, characterized by an abrupt transition between wildtype and aberrant expression, using the 10% cutoff.^28^, ^29^, ^30^ These cases were mainly characterized by a combination of p53 overexpression and a wildtype pattern (Figure 5A,B). Other cases showed wildtype and null patterns (Figure 5C) or a mixture of two mutant patterns and a wildtype pattern (Figure 5D). There was no difference in histologic features among parts of heterogeneously staining tumour cells. *TP53* mutations were identified in all five TNBC cases with subclonal p53 staining (Table 3). Two (cases 1 and 3) with a mixture of overexpression and wildtype patterns harboured missense mutations at the DBD. Other patterns (cases 2, 4, and 5) harboured frameshift or nonsense mutation located at the NLS, NES, or PRD.

**Table 3:** Subclonal p53 immunostaining patterns and *TP53* mutations

Case no.	Histologic type and grade	P53 immunostaining pattern and proportion	TP53 mutation	Mutation type	Domain	Hotspot	VAF	Nucleotide change	Amino acid change	LOH
1	IBC‐NST, G2	Overexpression (30%) and wildtype (70%)	Present	Missense	DBD	Yes	0.14	c.711G > A	p.Met237Ile	LOH
2	IBC‐NST, G3	Overexpression with cytoplasmic staining (20%) and wildtype (80%)	Present	Frameshift deletion	NLS	No	0.32	c.902del	p.Pro301GlnfsTer44	LOH
3	IBC‐NST, G2	Overexpression (15%) and wildtype (85%)	Present	Missense	DBD	Yes	0.17	c.524G > A	p.Arg175His	LOH
4	IBC‐NST, G3	Null pattern (90%) and wildtype (10%)	Present	Frameshift deletion	NES	No	0.38	c.1028del	p.Glu343GlyfsTer2	LOH
5	IBC‐NST, G3	Overexpression (40%), null pattern (30%), and wildtype (30%)	Present	Nonsense	PRD	No	0.13	c.281C > A	p.Ser94Ter	LOH

IBC‐NST, invasive breast carcinoma of no special type; G, grade; LOH, loss of heterozygosity; VAF, variant allele frequency.

## Discussion

While p53 IHC has been a useful surrogate marker for *TP53* mutation, its correlation with specific *TP53* mutations in TNBC has not been well understood yet. Its effectiveness in predicting mutation subtypes or functional properties remains unclear. Our findings indicate that an overexpression pattern of p53 IHC can reliably predict missense or in‐frame mutations and that it is significantly associated with GOF or DNE properties. Conversely, a p53 null pattern strongly indicates the presence of nonsense, frameshift, or splicing mutations, typically with NE‐GOF characteristics. Given these predictive capabilities, p53 IHC could be incorporated into clinical research designs to improve the selection of patients for therapies targeting specific *TP53* mutations.

The p53 overexpression pattern showed a high PPV for predicting *TP53* missense or in‐frame mutations in the DBD. These findings aligned with previous studies conducted on endometrial or ovarian carcinomas.^8^, ^10^, ^27^, ^31^, ^32^ It was noteworthy that all GOF mutations in our dataset exhibited p53 overexpression patterns. A GOF mutation may result in increased levels of both mutant p53 mRNA and protein, leading to accumulation of degradation‐resistant mutant p53 in the tumour nucleus.^26^, ^33^ These findings implicate a significant role of p53 IHC in predicting GOF mutations. The GOF of mutant p53 has been implicated in tumorigenesis, cancer invasion, and metastasis.^33^ Recent evidence indicates that GOF mutations are associated with poor prognosis in malignancies.^26^, ^33^ Various therapeutic strategies targeting mutant p53 with GOF have been proposed, several of which have advanced to clinical trials.^34^, ^35^ Our results suggest that the p53 IHC overexpression pattern could serve as a screening tool to predict poor prognosis or identify candidates for future targeted therapies.

Compared to the p53 overexpression pattern, the null pattern consistently showed nonsense, frameshift, or splicing mutation with a high predictive value. Therefore, the null pattern in TNBC represents a unique molecular characteristic, distinct from cases with wildtype p53. In line with findings from other solid tumours, the p53 null pattern in TNBC should not be simply classified as ‘p53‐negative’ based on usual cutoffs (e.g. less than 1% or 10% expression commonly used in breast cancer).^10^, ^36^, ^37^

Cytoplasmic p53 expression has been reported to be occurring in only 2%–3% of tumours, with varying definitions across studies.^8^, ^10^, ^31^ We considered cytoplasmic expression in over 80% of tumour cells as aberrant cytoplasmic expression, consistent with criteria from previous results of endometrial carcinoma.^32^ In this study, all cases with aberrant cytoplasmic expression were associated with nonsense or frameshift mutations in the C‐terminal side. Mutations at the C‐terminal end of p53 (residues 311–367) often lead to nontetrameric structures (monomers or dimers) that aberrantly accumulate in the cytoplasm, while functional tetramers localize to the nucleus.^10^, ^38^ The mechanisms underlying the simultaneous nuclear and cytoplasmic accumulation of mutant p53 remain unclear; however, this cytoplasmic expression may hold distinct molecular significance independent of concurrent aberrant nuclear expression.

We observed a subclonal p53 pattern in 4% of the TNBC cases. Their mutation types corresponded to areas of the aberrant p53 staining patterns observed. Recent studies on endometrial carcinoma have reported that cases exhibiting a subclonal p53 IHC pattern often harbour *TP53* mutations, supporting that this pattern represents true mutant subclones.^28^, ^32^ Our findings also indicate that a subclonal p53 staining pattern might represent a mutant‐type expression pattern, although the clinicobiologic significance of the cutoff has not been defined yet. The mechanism behind wildtype staining in *TP53*‐mutated tumour cells might be explained by the absence of LOH, although we sequenced the entire tumour area without distinguishing each component showing subclonal expression.

Upon reviewing the 10 cases with aberrant p53 expression where molecular testing failed to detect *TP53* mutations, we identified two cases with intratumoural heterogeneity. In these cases, less than 5% of cells showed p53 overexpression, which might have been undetected by sequencing. This discrepancy in the null pattern might be due to large *TP53* deletions undetectable by sequencing variant allele frequency. However, all cases showed LOH with shallow copy number alterations, and we could not determine the reason. In the case with *TP53* mutation but wildtype p53 expression, there was a splicing mutation with a variant allele frequency of 0.42, but it exhibited a wildtype pattern on p53 IHC. This type of discrepancy can be observed in splicing mutations or truncation mutations at the C‐terminal stop‐gain, resulting in a detectable but nonfunctional p53 protein that mimics the wildtype staining pattern.^27^

Mutant p53 proteins interact diversely with cellular components, resulting in both LOF and GOF effects.^39^ Some *TP53* mutations act dominantly negative, with mutant p53 disrupting wildtype p53 activity. This leads to loss of tumour‐suppressive function while gaining tumorigenic GOF properties. In our study, all DNE mutations showed p53 overexpression, with most also exhibiting GOF characteristics. These findings suggest that DNE mutations elevate mutant p53 levels in tumour cells, increasing the risk of inactive p53 complexes that impair wildtype function. However, DNE remains challenging to characterize, as mutant p53 effects on wildtype vary by tissue and context.^38^

In breast cancer research, a definitive cutoff value for p53 nuclear overexpression has not been established yet. The threshold for assessing p53 overexpression varies widely, from 1% to 90%.^40^ While a 10% cutoff has been suggested for breast cancer based on *TP53* mutations, recent studies report that overexpression in at least 80% of tumour cells significantly correlates with *TP53* mutations, although this arbitrary cutoff requires further validation.^15^, ^16^, ^41^, ^42^, ^43^ In our study, we identified 80% as the optimal cutoff for predicting *TP53* mutations and associating mutant p53 functionality in TNBC. However, this cutoff may not apply to all breast cancer subtypes, requiring further research in luminal and HER2‐enriched subtypes.

Limitation may arise from interpreting cytoplasmic expression of p53, as it can be complicated by potential overlap with nuclear expression and variations in IHC performance. To reduce these ambiguities, we used a high cutoff of 80% staining area for cytoplasmic expression based on the prior study.^32^ Nonetheless, additional studies are required to elucidate the significance of cytoplasmic expression, even in cases where it is focal. The limited progression events (six cases) and short follow‐up (median: 24 months) in our predominantly early‐stage cohort hindered detailed prognostic analysis of *TP53* mutation types. Ongoing efforts to expand the cohort, extend follow‐up, and include more high‐stage cases will enable robust analyses and validation of the prognostic value of *TP53*.

In conclusion, our study introduces newly defined criteria for interpreting p53 IHC expression in TNBC. These criteria could enable the prediction of *TP53* mutation types and their functional implications, providing valuable insights for future clinical research and potentially improving stratification of treatment options for patients.

## Author contributions

M.K., A.L., and J.K. designed the study. M.K. and M.L. collected patient materials, while B‐O.C., W.‐C.P., and J.L. provided the patient data necessary for this collection. M.K., M.L., A.L., and J.K. reviewed the slides and manually quantified the immunoexpression. B.‐O.C., W.‐C.P., and J.L. analysed and interpreted the patient data. J.K. provided the sequencing data. M.K and M.L performed the statistical analyses and interpreted the data. M.K. wrote the article and J.K. revised it. All authors read and approved the final article.

## Funding information

This study was supported by Research Fund of Seoul St.Mary's Hospital, The Catholic University of Korea (M.K., No. ZC22TISI0880) and Basic Science Research Program through the National Research Foundation of Korea (NRF) funded by the Ministry of Education (J.K., No. 2021R1I1A1A01043754).

## Conflict of interest

All authors declare no financial or nonfinancial competing interests.

## Supporting information


Data S1.

## Data Availability

The datasets used and/or analysed during the current study are available from the corresponding author on reasonable request.
