# Supplementary material for: Correlating p53 immunostaining patterns with somatic TP53 mutation and functional properties of mutant p53 in triple‐negative breast cancer
Source: Histopathology. 2025 Mar 31;87(2):299–309. doi: 10.1111/his.15453 (PMC12232215; doi:10.1111/his.15453)
Supplement: Supplementary file 1 — Data S1. [file HIS-87-299-s001.docx]

**Method S1. Ancillary testing procedures for TNBC confirmation**

ER (clone SP1; cat. no. 790-4324; Roche/Ventana, ready to use), PR (clone 1E2; cat. No. 790-4296; Roche/Ventana, ready to use), and HER2 (clone 4B5; cat. No. 790-4493; Roche/Ventana, ready to use). Additional HER2 silver *in situ* hybridization (SISH), when necessary, was conducted using the VENTANA HER2 Dual ISH DNA Probe Cocktail.

**Method S2. P53 immunohistochemistry and tissue processing protocols**

Tissue acquisition to fixation time was minimized to within 2 hours. Samples were then fixed in 10% neutral buffered formalin (NBF) for 6 – 72 hours and sliced at 5 mm intervals. Sections of 4-µm-thick were obtained from paraffin-embedded blocks, deparaffinized in xylene, and rehydrated through a graded series of ethanol. P53 IHC was performed for 113 TNBC cases with DO7 antibody (cat. No. 800-2912; Roche Diagnostics, IN, USA; ready to use) and a Leica Bond III system (Leica Biosystems, Inc., Buffalo Grove, IL, USA) following the manufacturer’s protocol.

**Method S3. Whole exome sequencing, copy number, and loss of heterozygosity analyses**

Whole exome sequencing was conducted for formalin-fixed, paraffin-embedded tissue blocks of a representative normal and tumor slide for the 113 TNBC cases. Paired-end sequences were generated with a NovaSeq6000 (Illumina, San Diego, CA, USA) platform following the manufacturer's protocols. The sequencing quality was checked using FastQC. Sequence reads were aligned to human genome assembly hg19 (GRCh37) using Burrows-Wheeler Aligner (BWA MEM, http://bio-bwa.sourceforge.net/), specifically the BWA-MEM algorithm.

Paired normal and tumor sequence reads were aligned and processed together to identify somatic *TP53* mutations. MuTect2 was used to detect somatic mutations. Mutations were filtered using the filtration tool of the Genome Analysis Toolkit (GATK). Functional annotation of filtered mutations was performed with SnpEff. Interpretation was performed with dbSNP and SNPs from the 1000 Genome Project. Further annotation with databases including ESP6500, ClinVar, dbNSFP, and American College of Medical Genetics and Genomics (ACMG) information was performed using an in-house program and SnpEff. VCF files of somatic mutations were processed to MAF files using the vcf2maf program. The ‘maftools’ R package was used to filter out synonymous mutations.

Copy number analysis of *TP53* was performed using GISTIC 2.0 (Genomic Identification of Significant Targets in Cancer, RRID:SCR_000151) applied to segmentation files obtained from Sequenza analysis. The analysis was undertaken to pinpoint significant loci of somatic CNVs. The analysis was configured with a focal length cutoff of 0.5 to focus solely on focal alterations. A confidence level of 0.9 was set to ensure high reliability of findings, along with a q-value threshold of 0.05 to distinguish between significant and non-significant results. Additionally, the X chromosome was excluded to eliminate variations related to sex chromosomes. The algorithm calculated G-scores for each region, taking into account the amplitude of copy number changes and their frequency across the sample set. Subsequently, it categorized these changes into homozygous deletions, heterozygous deletions, low-level gains, and amplifications.

To characterize the LOH at the *TP53* locus, the GenomicRanges package in R was employed to process segmented CNV data from whole-exome sequencing. We annotated segmented data for traceability and determined LOH status for each segment by assessing the absence of heterozygosity. This binary information was then integrated into a dataset. Specifically, segments overlapping with the TP53 gene on chromosome 17, indicating LOH at the *TP53* locus, were identified. These data were compiled into a *TP53*-specific LOH dataset for further analysis. If LOH occurred without a change in copy number in *TP53*, it was designated as copy neutral LOH.


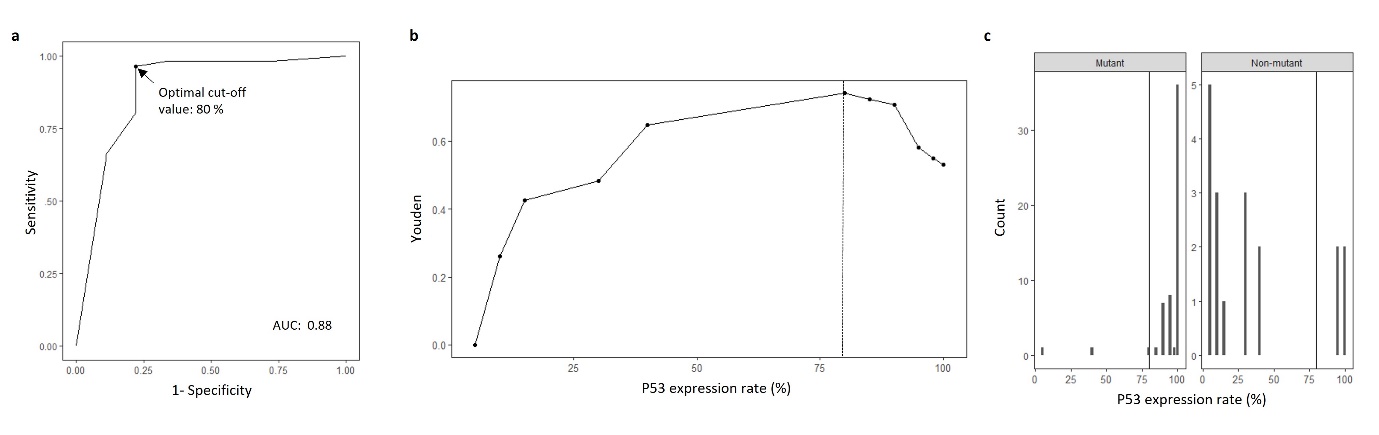


**Fig. S1. Evaluation of an optimal cutoff value for p53 nuclear expression.** (a) A receiver operating characteristic (ROC) curve with a cut-off value of 80% for p53 nuclear expression shows an area under the curve (AUC) of 0.88. (b) The Youden index values increase with the rising rate of p53 nuclear expression, reaching the optimal value at 80% (indicated by the dashed line). (c) The histogram compares the distribution of p53 nuclear expression rates between cases with TP53 mutations (labeled as "Mutant") and those without TP53 mutations (labeled as "Non-mutant") with a reference line at 80%.

# Table S1. Baseline characteristics of patients with triple-negative breast cancer

| Variables | Patients, n (%) |
| --- | --- |
| Age, years |  |
| Median (range) | 53 (28 – 83) |
| Histological type |  |
| Invasive breast carcinoma, NST | 97 (85.8) |
| Carcinoma with apocrine differentiation | 5 (4.4) |
| Metaplasitc carcinoma | 3 (2.7) |
| Carcinoma with medullary feature | 2 (1.8) |
| Others ^a^ | 6 (5.3) |
| Histological grade |  |
| Grade 1 | 1 (0.9) |
| Grade 2 | 19 (16.8) |
| Grade 3 | 93 (82.3) |
| TNM Stage |  |
| Ⅰ | 36 (31.9) |
| Ⅱ | 71 (62.8) |
| Ⅲ | 6 (5.3) |
| Ⅳ | 0 (0) |
| Total | 113 (100) |

^a^ Others: Invasive lobular carcinoma, adenoid cystic carcinoma, invasive micropapillary carcinoma.

NST, no special type

# Table S2. Gain-of-function properties of *TP53* mutations and associated literature references

| **Variant** | **Gain-of-function** | **No. of cases^b^** | **Hotspot** | **No. of references^c^** |
| --- | --- | --- | --- | --- |
| p.R175H | TA, p73i, DR, GA, TRANSF+ | 7 | yes | 53 |
| p.R273H | TA, p73i, DR, GA, TRANSF+ | 5 | yes | 56 |
| p.R273C | TA, p73i, DR, TRANSF+ | 2 | yes | 13 |
| p.H179Y | TA, p73i, DR, GA, TRANSF+ | 2 | Yes | 5 |
| p.R280K | TUMOR+ | 2 | yes | 1 |
| p.Y220C | p73i, DR | 2 | yes | 4 |
| p.G245C | Other (Hsc70 binding)^a^ | 1 | yes | 1 |
| p.M237I | TA, TRANSF+ | 1 | yes | 3 |
| p.G266E | p73i | 1 | yes | 1 |
| p.V173L | TA, p73i, drug resistance, TRANSF+ | 1 | yes | 7 |
| p.R280T | GA | 1 | yes | 2 |
| p.G245D | p73i, drug resistance, TRANSF+ | 1 | yes | 3 |
| p.R282G | p73i | 1 | yes | 1 |
| p.Y126C | TA | 1 | yes | 1 |
| p.C176F | TA, GA | 1 | yes | 1 |
| p.C242Y | p73i, drug resistance | 1 | yes | 2 |
| p.R156P | p73i, drug resistance, TRANSF+ | 1 | no | 4 |

TA, transactivation of genes repressed by wild-type p53; p73i, ability to counteract p73 (a p53 homologue) activity when both are expressed in a cell system (p73 interference); TUMOR+, confer tumorigenic property (in nude mice) to transfected cells; DR, confer resistance to a cytotoxic drug (Drug resistance); GA, increase growth rate (Growth advantage); TRANSF+, ability to cooperate with *RAS* or another transformant oncogene, such as HPV E7, in the transformation of primary cells.

^a^ alteration of mutant p53 stability and activity by impairing regulator, HSC70

^b^ Number of cases displaying gain-of-function properties analyzed in this study

^c^ Number of published references supporting the fain-of-function properties of the *TP53* mutation
